# Intravital imaging of osteocytes in mouse calvaria using third harmonic generation microscopy

**DOI:** 10.1371/journal.pone.0186846

**Published:** 2017-10-24

**Authors:** Danielle Tokarz, Richard Cisek, Marc N. Wein, Raphaël Turcotte, Christa Haase, Shu-Chi A. Yeh, Srinidhi Bharadwaj, Anthony P. Raphael, Hari Paudel, Clemens Alt, Tzu-Ming Liu, Henry M. Kronenberg, Charles P. Lin

**Affiliations:** 1 Advanced Microscopy Program, Center for Systems Biology and Wellman Center for Photomedicine, Massachusetts General Hospital, Harvard Medical School, Boston, Massachusetts, United States of America; 2 Department of Physical and Chemical Sciences, University of Toronto, Mississauga, Ontario, Canada; 3 Endocrine Unit, Massachusetts General Hospital, Harvard Medical School, Boston, Massachusetts, United States of America; 4 Dermatology Research Centre, The University of Queensland Diamantina Institute, Translational Research Institute, Brisbane, Queensland, Australia; 5 Institute of Biomedical Engineering and Molecular Imaging Center, National Taiwan University, Taipei, Taiwan. Faculty of Health Sciences, University of Macau, Macau SAR, China; Augusta University Medical College of Georgia, UNITED STATES

## Abstract

Osteocytes are the most abundant cell in the bone, and have multiple functions including mechanosensing and regulation of bone remodeling activities. Since osteocytes are embedded in the bone matrix, their inaccessibility makes *in vivo* studies problematic. Therefore, a non-invasive technique with high spatial resolution is desired. The purpose of this study is to investigate the use of third harmonic generation (THG) microscopy as a noninvasive technique for high-resolution imaging of the lacunar-canalicular network (LCN) in live mice. By performing THG imaging in combination with two- and three-photon fluorescence microscopy, we show that THG signal is produced from the bone-interstitial fluid boundary of the lacuna, while the interstitial fluid-osteocyte cell boundary shows a weaker THG signal. Canaliculi are also readily visualized by THG imaging, with canaliculi oriented at small angles relative to the optical axis exhibiting stronger signal intensity compared to those oriented perpendicular to the optical axis (parallel to the image plane). By measuring forward- versus epi-detected THG signals in thinned versus thick bone samples *ex vivo*, we found that the epi-collected THG from the LCN of intact bone contains a superposition of backward-directed and backscattered forward-THG. As an example of a biological application, THG was used as a label-free imaging technique to study structural variations in the LCN of live mice deficient in both histone deacetylase 4 and 5 (HDAC4, HDAC5). Three-dimensional analyses were performed and revealed statistically significant differences between the HDAC4/5 double knockout and wild type mice in the number of osteocytes per volume and the number of canaliculi per lacunar surface area. These changes in osteocyte density and dendritic projections occurred without differences in lacunar size. This study demonstrates that THG microscopy imaging of the LCN in live mice enables quantitative analysis of osteocytes in animal models without the use of dyes or physical sectioning.

## Introduction

Osteocytes are the most abundant cell in bone, forming a large surface area network referred to as the osteocytic lacunar-canalicular network (LCN). Through the LCN, osteocytes maintain intercellular communication of signals that regulate both bone formation and bone resorption. However, our knowledge about the structure and mechanical properties of the LCN remains incomplete due to the challenging assessment of this network. Existing high resolution imaging techniques to visualize the osteocyte LCN *ex vivo* include light microscopy, scanning electron microscopy, transmission electron microscopy, confocal microscopy, X-ray computed tomography, transmission X-ray microscopy, and ptychographic computed tomography [1]. These techniques require *ex vivo* sample preparation such as thin sectioning of embedded or cryopreserved tissues or plastic-embedded thick tissue sectioning and serial milling [2]. Three-dimensional imaging can be achieved with thin serial sections of tissue; however, digital reconstruction is necessary and in most cases artefacts including morphological distortion and sample loss are often unavoidable [2]. Three-dimensional imaging can be more easily achieved with plastic-embedded thick tissue sections; however, harsh embedding conditions and tissue sectioning artefacts often limit the representation of the images to the *in vivo* condition [2].

These limitations can be overcome by imaging the LCN *in situ*, either in explanted bone samples or *in vivo*, using confocal microscopy techniques. For example, the LCN has been imaged in explanted bone by infusing the animal with dyes such as calcein and alizarin red [3–7] prior to sacrificing the animal and dissection. Transgenic animal models with fluorescent reporter proteins of gene expression have also been applied to study osteocytes [1, 8]. For instance, transgenic mice expressing the *topaz* variant of GFP under the control of the osteocyte-selective dentin matrix protein-1 (Dmp1) promoter [9] have been used to demonstrate the motion of the osteocytic dendrites during time-lapse imaging of the explanted mouse calvarium [10]. Further, two-photon excitation fluorescence (2PF) microscopy has been used to visualize the LCN by *in vivo* calcein [11] and rhodamine B staining [12]. However, fluorophores have a tendency to photobleach, especially when high illumination power is needed to visualize weak fluorescence signals such as those associated with canaliculi.

Another strategy that recently evolved in microscopic imaging is the use of parametric processes, such as second harmonic generation (SHG) and third harmonic generation (THG), which do not require absorption and therefore, do not induce photochemical reactions leading to photobleaching. SHG and THG are nonlinear optical processes that depend on the simultaneous arrival of two or three photons to generate an output photon with exactly one half (SHG) or one third (THG) the wavelength of the incoming photons. Although any multiphoton microscopes equipped with femtosecond lasers are in principle capable of SHG and THG imaging, in practice a laser emitting at wavelengths of 1200 nm or longer and a microscope with optics optimized for such long wavelengths are required in order for the THG signal to be detectable within the visible range (greater than 400 nm). As in other nonlinear optical processes, SHG and THG signals fall off sharply away from the laser focus. Consequently, by scanning the laser focus in a 2-dimensional plane, an optical section is automatically produced without the need for confocal detection. The SHG and THG signal intensities are determined by the nonlinear optical properties of the molecules constituting the material and the structural symmetry of the material. SHG is described by a second-order nonlinear optical susceptibility tensor (*χ*^(2)^) which is a function of the constituent molecules ability to produce SHG as well as their organization into a structure with noninversion symmetry. For example, intense SHG occurs in collagenous materials [13, 14], such as bone [11, 15]. THG is produced at an interface where a change in refractive index or third-order nonlinear optical susceptibility (*χ*^(3)^) is present [16]. Therefore, biological membranes and multilayer structures are readily visualized with THG microscopy [17]. In addition, SHG and THG signals can differ when detected in the forward- or epi-direction with respect to the optical axis [18, 19].

In this study, the osteocyte LCN was imaged using THG microscopy *in vivo*. The origin of THG signals was interrogated by comparing THG images with three-photon fluorescence (3PF) images of SR101 labeled mice, and with 2PF images of transgenic GFP mice. Notably, the osteocyte LCN was detected in the THG images as bright structures (positive contrast) against a dark background of the surrounding bone. This was opposite to SHG imaging of bone: the lacunae were seen as dark voids (negative contrast) embedded in a bright background generated by the collagen in the bone [11]. The negative contrast of SHG microscopy rendered the canaliculi difficult to be detected: the empty structures tended to be masked by positive signals from the surrounding bone, as the sizes of these structures approached the optical resolution limit, whereas the sub-resolution structures could be detected by THG even if their sizes could not be determined accurately. Forward- and epi-emitted THG was compared, and the THG intensity emitted from canaliculi was measured as a function of their angle from the optical axis. The live imaging technique was subsequently demonstrated for visualizing differences between the osteocytes of wild type (WT) and histone deacetylase 4 and 5 (HDAC4, HDAC5) double knockout mice (HDAC4/5 DKO). Mice in which these two genes are deleted in osteocytes show high sclerostin expression, disorganized “woven” cortical bone, and high osteocyte density [20]. Sclerostin, which is encoded by the SOST gene, is important to study because it inhibits osteoblastic bone formation and is therefore a drug target for osteoporosis [21]. Three-dimensional analysis of the number osteocytes per volume, the lacunar volume and surface area as well as the number of canaliculi per lacunar surface area were performed with THG imaging, revealing variations, and demonstrating the capability of THG microscopy for *in vivo* LCN studies.

## Materials and methods

### *In vivo* mouse preparation

All animal experiments were performed in compliance with institutional guidelines and approved by the Massachusetts General Hospital Institutional Animal Care and Use Committee. The mice used in this study consisted of WT C57BL/6J mice (Jackson Laboratory), transgenic 2.3ColGFP mice generously provided by Dr. David Rowe (University of Connecticut) which express the Green Fluorescent Protein in osteoblasts and osteocytes, and HDAC4/5 DKO mice [20] with the following genotype: HDAC4f/f; HDAC5-/-;DMP1-Cre (9.6 kB promoter [22]).

To image the osteocytes in the calvaria, WT, 2.3ColGFP, and HDAC4/5 DKO mice were anesthetized with isoflurane gas, and placed in a custom 3D printed and heated mouse holder. An incision was made on the top of the skull. Skin was partly detached exposing the periosteum and the calvarial bone [23]. A microscope cover glass holder was used to hold a microscope cover glass over the dissected area, which was hydrated with phosphate-buffered saline (PBS). The imaging locations were consistently chosen in regions 3, 4, 5 and 6 [24], 100–300 μm away from the coronal and central veins, while imaging depths were chosen, starting from ~10 μm below the bone surface. Although osteocytes in the calvaria of mice were imaged due to ease in accessibility, imaging of the tibia or femur is possible, using a custom mouse holder as described by Sano et al. [11]. Mice were euthanized immediately after imaging experiments by cervical dislocation.

For 3PF imaging of osteocytes labeling was performed with the dye sulforhodamine 101 (SR101, Sigma Aldrich, St. Louis, MO). SR101 is commonly used for fluorescence microscopy imaging of astrocytes in the brain and the spinal cord. About 20 μL of 10 mM SR101 dissolved in PBS was applied to the periosteum and allowed to diffuse for several minutes. 3PF imaging was performed with 1700 nm wavelength excitation.

### Nonlinear optical microscopy setup

A turn-key laser with a 5 MHz repetition rate, 370 fs duration pulses and 1550 nm wavelength (FLCPA-01CCNL41, Calmar) was coupled to a 60 cm long polarization maintaining large mode area single mode photonic crystal fiber with a 40 μm diameter core (LMA40, NKT Photonics, Birkerød, Denmark). A pulse energy of 140 nJ in the fiber generated a soliton at 1700 nm via a soliton self-frequency shift measured with a laser spectrometer (waveScan USB, APE Angewandte Physik & Elektronik GmbH, Berlin, Germany). The soliton had a pulse energy of 23 nJ and a pulse width of 60 fs as measured with an autocorrelator (REEF AA-20DD, Del Mar Photonics, San Diego, CA). The soliton was spectrally filtered using a 1600 nm long pass filter (84–680, Edmund Optics, Barrington, NJ). At lower fiber input laser powers, compressed 1550 nm pulses were generated with 60 fs and used for imaging. Using a bismuth borate (BiBO) crystal (Newlight Photonics, Toronto, Canada), a secondary light path was made such that 1700 nm could be frequency doubled to 850 nm yielding a pulse energy of 12 nJ for two-photon excitation.

**Fig 1:**
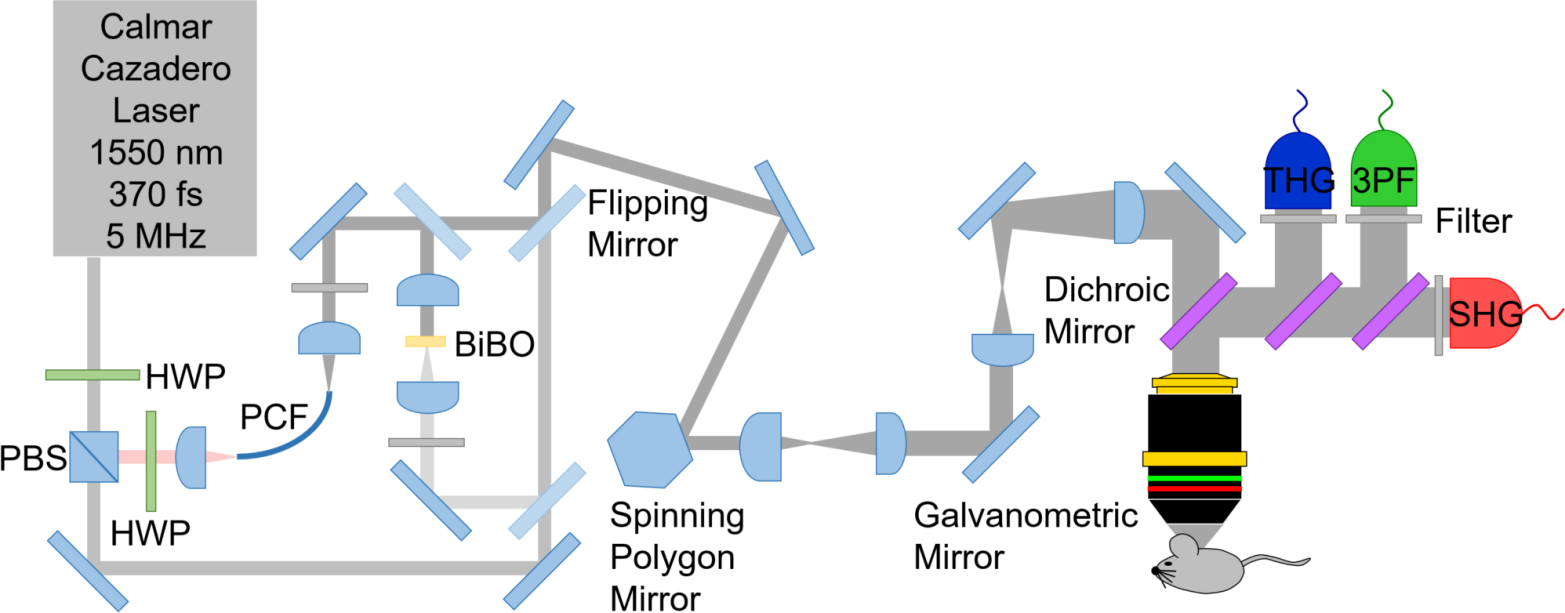
A schematic of the laser microscope for three-photon imaging using 1550 nm and 1700 nm. Second and third harmonic generation (SHG and THG) as well as three photon excitation fluorescence (3PF) are collected while the laser is scanned with a polygonal and galvanometric mirror pair. HWP represents half-wave plates, PCF represents a polarization maintaining large mode area single mode photonic crystal fiber for generation of 1700 nm, while BiBO represents a bismuth borate crystal for frequency doubling of 1700 nm.

All wavelengths (850 nm, 1550 nm, and 1700 nm) were coupled to an in-house laser scanning microscope. The microscope setup shown in Fig 1 consisted of a polygonal laser scanner (DT-36-290-025, Lincoln Laser, Phoenix, AZ) for the fast axis and a galvanometric scanning mirror for the slow axis (6240H, Cambridge Technology, Bedford, MA) similar to previous imaging platforms [25], achieving 15 frames per second with 500 × 500 pixels. An oil immersion objective lens (1.05 NA, UPLSAPO 30×SIR, Olympus, Waltham, MA) was used for imaging. Mineral oil (Sigma Aldrich) was used as the immersion oil due to its high transmission at 1550 nm and 1700 nm. During imaging with 2PF at 850 nm, a pulse energy of 3 nJ was used at the sample, while 3PF and THG imaging required 10 nJ of 1700 nm and 1550 nm. Alternatively, THG can be obtained in a standard two-photon microscope equipped with an optical parametric oscillator to extend the wavelength to >1200 nm, and exchanging the optics for the higher wavelength compatibility.

As shown in Fig 1, SHG, THG and 3PF signals with 1550 nm excitation were collected using photomultiplier tubes (PMT) (SHG: R7600U-20, Hamamatsu, Bridgewater, NJ, THG and 3PF: R7600U-300, Hamamatsu). These signals were collected in the epi-direction through the objective lens, enabling imaging of thick tissue samples. SHG signal, which visualizes the bone, was collected in order to verify the imaging location in the calvarium. 3PF signal from a dye (SR101) was collected in order to verify the origin of THG. SHG, THG and 3PF signals were separated with dichroic mirrors from the laser (DMLP950R, Thorlabs Inc., Newton, NJ) and from each other (FF526-Di01, Semrock, Rochester, NY, and 69–904, Edmund Optics), and then filtered (SHG: FF01-780/12, Semrock, THG: 65–153, Edmund Optics, 3PF: BLP01-514R-25, Semrock). For THG detection with 1700 nm excitation, a different filter was used (86–985, Edmund Optics). SHG, THG and 3PF analog signals were digitized at the laser repetition rate using the laser sync output as a pixel clock with an eight bits frame grabber card (Snapper-PCI-8/24, Active Silicon, Severna Park, MD).

For two-photon excitation fluorescence microscopy imaging with 850 nm excitation, the 850 nm light after the doubling crystal was filtered (D850/300, Chroma, Burlington, VT). 2PF signal was separated from the laser with a dichroic mirror (69–904, Edmund Optics) and directed to the detector using another dichroic mirror (FF705-Di01, Semrock). The 2PF signal was filtered (FES0800, Thorlabs Inc. and HQ520/120 M, Chroma) and collected using the same PMT used for THG detection in Fig 1. The 2PF contrast was used to verify the location of osteocyte cells in a transgenic 2.3ColGFP mouse.

The directionality of THG emission from osteocytes was investigated. Unlike fluorescence which is emitted isotropically, THG emission in most cases occurs in the forward direction due to momentum conservation [26], referred to as forward-directed THG. The initial directionality of the signal is an important parameter for proper intensity quantifications. Forward- and backward-directed signals of similar intensities would appear with different intensities in the epi-detection channel since only a fraction of the forward-directed THG would reach the epi-detector via backscattering. Phase matching conditions in combination with the coherent addition of reflections can result in changes in directionality of THG signal [27, 28]. Backward-directed THG was experimentally realized in a water wedge study [28], as well as, in thin films of ZnO and lipid enclosed quantum dots of CdSe and Fe_3_O_4_ [19]. In the present experiment, the *in vivo* directionality of THG was inferred by imaging a dissected calvarial bone, which was thinned to ~20 μm. This thickness reduces the backscattered forward-emitted THG, since this thickness is less than one reduced scattering mean free path, while simultaneously, allowing a measure of direct forward-emitted THG.

The thinned bone situated between two cover glasses was imaged using the setup shown in Fig 1, which was modified by placing a custom 0.8 NA collection objective lens, THG filter and THG PMT in the forward direction, directly after the bone sample in the beam path. In order to compare forward- and epi-detected signals, the same PMT and optical filter were used in both collection orientations. Further, the THG signal collection efficiency in forward- and epi-orientations was normalized with data from imaging 3PF of 3.0 μm red fluorescent beads (R0300, Duke Scientific Corporation), and corrected for the variation in NA. It was ensured that a broad range dichroic mirror (FF801-Di02-25 × 36, Semrock) was used for both THG and 3PF imaging which had similar reflections at both THG and 3PF emission wavelengths (98.5% and 98.9% reflection, respectively). Additionally, for calibration, a 3PF filter (BLP01-514R-25, Semrock) was used, which blocked THG emitted from the beads. The relative intensity of THG from the thinned bone sample was collected with the detector in the forward and epi geometries in order to reveal the emission orientation of the THG signal.

Bone thinning was performed using another microscope setup similar to the microscope seen in Fig 1 and described elsewhere [29, 30]. Briefly, a turn-key 5 MHz repetition rate laser with 370 fs duration pulses and 1550 nm wavelength (FLCPA-01CCNL31, Calmar, Palo Alto, CA) was doubled to 775 nm using a 0.5 mm bismuth borate crystal (Newlight Photonics) and this wavelength was used for laser-induced plasma-mediated ablation. To thin the bone, clean ablation craters were induced under water immersion with 22 nJ pulse energy with 0.5 μm step size during continuous flushing with PBS at 10 mL/min. Bone thinning was monitored by SHG imaging between laser ablation treatments.

### Image processing

A systematic comparison of 2D images from 3PF, 2PF, and THG was performed using structural image cross correlation analysis (SCIA) using a custom LabVIEW (National Instruments Corp., Austin, TX) interface, which was previously described [31,32]. Briefly, the 3 images (A, B and C) were compared using a custom algorithm, pixel by pixel, after manual thresholding of the signal. The algorithm resulted in 7 images, denoted using ∩ which indicates the logical intersection, and ~ which indicates the logical not. The seven images were as follows: (1) A∩B∩C, signal in all three channels, (2) A∩B∩~C, overlap between channels A and B, but not C, (3) A∩C∩~B, overlap between channels A and C, but not B (4) B∩C∩~A, overlap between channels B and C, but not A, (5) A∩~B∩~C, pixels in channel A that are not correlated with B or C, (6) B∩~A∩~C, pixels in channel B that are not correlated with A or C, and (7) C∩~A∩~B, pixels in channel C that are not correlated with A or B. The 7 images are mutually exclusive, therefore, one color can be assigned to each image and there will be no overlap when all 7 images are shown together. The algorithm is advantageous compared to simple overlap because the colors do not mix, therefore, interpretation of the image is unambiguous and clear.

The THG intensity corresponding to the canaliculus tilt angle was measured from optical sections of the calvarial bone. The X and Y position of a bright spot along the canaliculus was noted in each optical section image, which were 1 μm apart. From these positions, the average tilt angle was calculated and plotted as a function of the average THG intensity of the bright spots in the slices. Analysis was performed on 34 different canaliculi.

Three-dimensional analysis of osteocytes was performed in ImageJ 1.50i (NIH, Bethesda, MD) and Imaris 7.4.2 (Bitplane Inc., South Windsor, CT). The number of osteocytes in a volume of 100 μm × 100 μm × 10 μm was manually counted by selecting this volume in ImageJ from optical sections and manually counting the number of osteocytes. Manual thresholding of the signal intensity from the lacunae was achieved in Imaris allowing the software to calculate the volume and surface area of individual lacuna. The number of canaliculi per osteocyte was measured by imaging osteocytes with an image size of 244 × 147 μm, manually thresholding the optical sections in Imaris and using the FilamentTracer function in Imaris to trace the canaliculi. Once the canaliculi were rendered, the number of canaliculi was manually counted.

## Results and discussion

### Origin of the THG image contrast in bone

**Fig 2:**
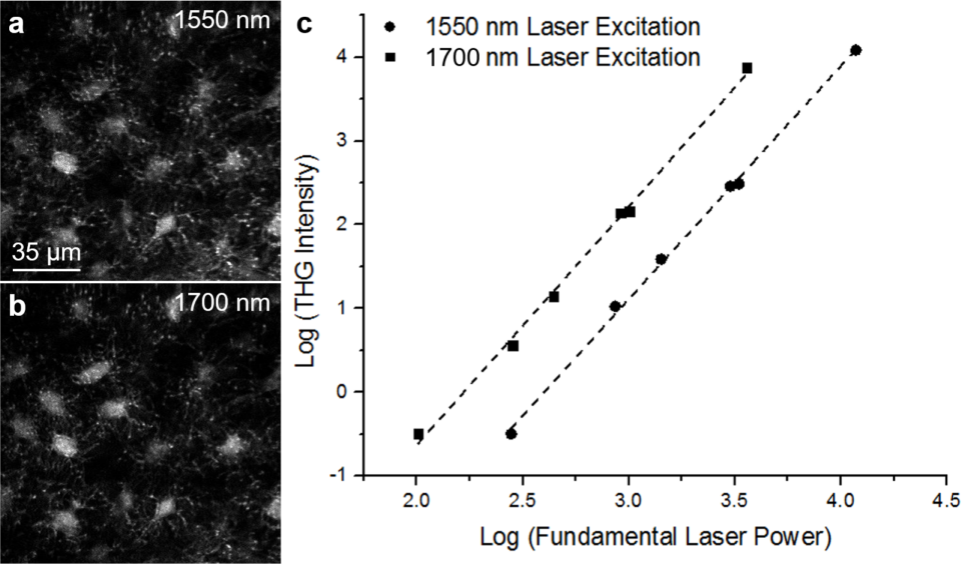
THG power dependency plot. THG images of osteocytes obtained at 1550 nm (a) and 1700 nm (b) excitation *in vivo* as well as a logarithmic plot (c) of the THG intensity of an osteocyte with fundamental laser power for both 1550 nm and 1700 nm excitation. The data was fit with a line corresponding to a slope of 2.8 ± 0.2 for 1550 nm excitation and 2.9 ± 0.2 for 1700 nm excitation.

The calvarium of a WT C57B6/J mouse was imaged with femtosecond laser pulses at either 1550 nm or 1700 nm. The epi-detected THG signal (collected through the same objective lens used for the laser excitation) arises from lacunae, the larger (micrometer) spaces inside bone that contain osteocyte cell bodies, as well as canaliculi, the small (sub-micrometer) channels that interconnect lacunae and contain osteocyte processes (Fig 2A and 2B). The THG intensity from osteocytes at various input laser powers at 1550 nm and 1700 nm was plotted (Fig 2C) and showed the expected power dependence.

Since THG cannot be obtained from homogeneous isotropic materials with normal dispersion [33], the observed signals result from heterogeneity of the medium containing interfaces consisting of changes in refractive index or *χ*^(3)^. Due to the fact that osteocytes and their processes are surrounded by interstitial fluid, it is likely that THG microscopy is visualizing two distinct heterogeneities: one between the bone and the interstitial fluid, and another between the interstitial fluid and the cell. In order to clarify the signal origin, 2PF, 3PF and THG signals were collected. The 2PF image was collected using a frequency-doubled 1700 nm beam, and a second scan was performed with 1700 nm excitation to obtain 3PF and THG signals.

Multicontrast imaging of osteocytes in the SR101-labeled transgenic 2.3ColGFP mouse was performed to clarify the origin of the THG signals. SR101 was used since it is known that when applied to intact calvarium, the dye passes directly through the bone and into the meninges within a short period of time, and it does not permeate the osteocytes [34]. In the current experiment, SR101 was applied to the skull of a live transgenic 2.3ColGFP mutant mouse after the skin was removed while the periosteum was left intact. This mouse strain was chosen because it expresses GFP in the osteocytes, allowing them to be visualized with 2PF contrast.

**Fig 3:**
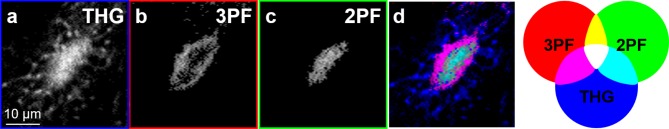
Images of a lacuna and canaliculi in the calvarial bone of a transgenic 2.3ColGFP mouse. THG (a) and 3PF of SR101 dye (b) were imaged with 1700 nm excitation while 2PF from GFP (c) was imaged with 850 nm excitation. A structural image cross-correlation analysis [31, 32] between 3PF, 2PF, and THG was performed (d).

A typical image of the THG signal from the lacuna and canaliculi present in the mouse calvarium is visualized in Fig 3A while 3PF signal from SR101 is shown in Fig 3B, and 2PF signal from GPF in the osteocyte cell is presented in Fig 3C.

The correlated image of the three contrasts (see Materials and methods) in the osteocyte (Fig 3D) shows that the 3PF signal from SR101 correlates with the outer edge of the osteocyte THG signal, but it does not correlate with 2PF signal from the osteocyte cell. Since SR101 is known not to bind to cellular structures present in osteocytes, it indicates that the 3PF signal from SR101 is representative of an area within a lacuna containing interstitial fluid. This is supported by the observation that the 2PF image localizing the osteocyte with GFP does not spatially correlate with the SR101 3PF signal. Therefore, the corresponding THG signal outlines the bone-interstitial fluid interface. Similarly, the THG signal at the canaliculi is expected to be dominated by the bone-interstitial fluid interface rather than the boundary of the osteocyte dendritic processes.

### Epi-THG versus forward-THG from the LCN

In order to further understand the origin of the epi-collected THG signals from the lacunae and canaliculi, the difference between forward-collected versus epi-collected THG signal of a thinned calvarial bone from a WT mouse was investigated *ex vivo*. This was done to determine whether the epi-collected THG from the lacunae and canaliculi primarily originates from direct backward-directed THG or from back-scattered forward-propagating signal, see Materials and methods section for further details. The bone was thinned to a thickness of ~20 μm as determined with SHG imaging. This dissected thin bone region allows collection of the THG in the forward direction. Additionally, this thickness is much less than one reduced scattering mean free path in bone needed for efficient backscattering to occur [35, 36], ~625 μm at 1550 nm, based on the reduced scattering coefficient (μ_*s*_*’*) of 16 cm^-1^ from human cranial bone [37]. From simulations, at 20 μm tissue thickness, < 5% of forward propagating photons are expected to be backscattered [38].

**Fig 4:**
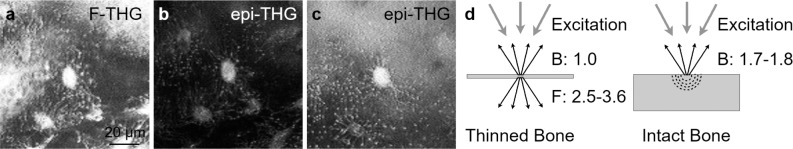
Forward-THG versus epi-THG from the LCN. Forward-THG (F-THG) (a) and epi-THG (epi-THG) (b) images of lacunae and canaliculi in thinned calvarial bone from a WT mouse taken with 1550 nm excitation and compared to an epi-THG image of the calvarial bone of WT mouse *in vivo* without bone thinning (c). The results are summarized in (d) showing that epi-collected THG is a combination of backwards-directed and backscattered forward-generated signals.

Typical THG signals from thinned bone were collected sequentially via forward- and epi-collection. As shown in Fig 4A and 4B, the lacunae and canaliculi are clearly visible in both modalities. The average THG intensity for bright regions along the canaliculi in the thinned calvarial bone with forward-collection was found to be 2.5 ± 0.3 times more intense than epi-collected THG, after normalization of the signal using 3PF and NA correction. Similarly, the average THG intensity for lacunae in the thinned calvarial bone with forward-collection was found to be 3.6 ± 1.4 times more intense than epi-collected THG. The epi-collected THG intensity of canaliculi and lacunae in thinned and thick (intact) bone was also compared. Imaging of the LCN nearest to the outer surface of the calvarium of a live WT mouse (Fig 4C) revealed a higher intensity of epi-collected THG as compared to the thinned sample. The THG intensity of live mouse canaliculi and lacunae was found to be 1.8 ± 0.4 and 1.7 ± 0.9 times greater, respectively, than canaliculi and lacunae in the thinned bone. These results, summarized in Fig 4D, show that the epi-collected THG from intact bone is a superposition of backward-directed and back-scattered forward-generated signals.

### Higher THG signal intensity from canaliculi oriented at small angles to the optical axis

**Fig 5:**
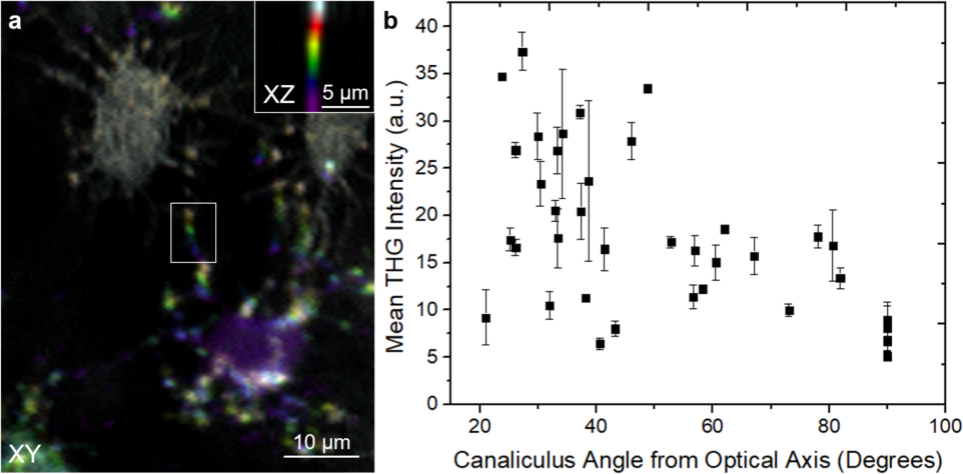
THG signal intensity from canaliculi oriented at a number of angles to the optical axis. From optical sections of epi-THG collected signal from a WT mouse *in vivo*, a summed image of 6 consecutive XY slices which are 2 μm apart in Z were colored in such a way that the top slice is white, second slice is red, third slice is yellow, fourth slice is green, fifth slice is blue and the bottom slice is purple (a). A summed image of 6 consecutive XZ slices of the area of the canaliculus outlined in a white box is shown in the inset of (a) demonstrating that the canaliculus is parallel to laser propagation which is along Z. The tilt angle between bright areas within a canaliculus was calculated and the corresponding average THG intensities were plotted (b).

During epi-THG imaging, which is essential for *in vivo* studies of the mouse calvarium, the canaliculi appear as two distinct structures, lines and bright spots (Fig 4). Canaliculi that are perpendicular to the optical axis appear as lines, while canaliculi at decreased angles to the optical axis appear as bright spots localized at the intersection between the imaging plane and the canaliculi. The latter can be most easily observed in Fig 5A, a 2D representation shown perpendicular to the optical axis (XY) for an en face view of the LCN. Here, six consecutive optical slices, each a different color, were summed along the optical axis. Likewise, a cross-sectional view parallel to the optical axis (XZ) is shown in the inset. Notably, canaliculi that are tilted out of the image plane toward the optical axis have significantly increased THG intensities as compared to canaliculi that are in the image plane, perpendicular to the optical axis.

From epi-collected THG images of a mouse *in vivo*, the canalicular intensities and angles to the optical axis were obtained (see Materials and methods). Kendall’s statistical correlation test was performed, confirming a positive correlation between canaliculus angle and THG intensity, with statistical factor τ = -0.47. As expected, there is a distribution of THG intensities at each angle (Fig 5B), likely because of the difference in diameters and surface roughness of the canaliculi, which reduce the correlation from τ = -1. However, it is clear from this graph that canaliculi at lower angles to the optical axis have higher THG intensities.

The observation that increased THG signal corresponds to canaliculi which are angled towards the optical axis is notable because according to the Gouy phase shift with a Hermite-Gaussian (HG_00_) beam, the forward-collected THG intensity at an interface should increase with increasing tilt of the interface from the optical axis when a high NA excitation objective lens and an equal or lower NA collection objective lens are used [39]. This has been previously demonstrated experimentally in at least two different experiments, during THG imaging of a fiber in index matching fluid [17] and during imaging of polystyrene beads [40, 41] where the diameters of these samples are larger than the laser point spread function. Intense THG signals are mostly observed at the top and bottom interfaces where the optical axis is perpendicular to these interfaces while much lower THG signals are observed at the sides of the fiber and beads where the optical axis is parallel to these interfaces [40, 41].

On the other hand, Olivier *et al*. demonstrated, using a vector field model, that the THG signal from a slab parallel to the optical axis is significantly greater than THG signal from a slab perpendicular to the optical axis, for slab thickness < 1 μm [42]. Since the far-field radiation pattern of forward-THG of a structure with interfaces parallel to the laser propagation occurs along directions at large angles away from the interface, as determined by phase mismatch [43], a high NA collection objective lens, higher than the excitation objective lens, is needed in order to detect the forward-THG signal from interfaces parallel to the optical axis due to the THG double lobe far-field radiation pattern [27]. Since in this work the signal is collected in the backwards geometry, it is plausible that the forward-generated THG at large angles away from the optical axis favors its detection in the epi-direction.

The increased THG of canaliculi oriented toward the optical axis may also occur due to an increased canalicular volume in the focus of the laser, especially considering canaliculi are not smooth. From electron micrographs of the canaliculi of osteocytes in mice humeri [44] and femur [45] as well as human femur [46], it appears that the canaliculi are corrugated structures. Further, from ultra-high voltage electron microscopy images of chick calvaria, images of reconstructed osteocytes show that the surfaces of the osteocyte cell body and processes are irregular as well [47]. Previously, it has been shown that corrugated nanowires have heterogeneous optical properties which lead to variations in THG signal along the nanowire [48]. Similarly, due to the corrugated nature of the canaliculus and osteocyte processes, in canaliculi that are increasingly parallel to the laser beam, many interfaces exist that are perpendicular to the optical axis, and thereby could emit THG. In the current imaging system, the point spread function was ~0.5 μm laterally and ~3.0 μm axially. Therefore, the corrugated canaliculi, being smaller than the point spread function, have a larger volume within the focus when they are oriented towards the optical axis, hence this geometry may result in larger THG signal, since THG is a volume effect [49].

### THG image contrast of the LCN in mouse calvaria demonstrates differences between WT and HDAC4/5 DKO mice

Finally, we asked if THG could be used to distinguish osteocyte phenotypes in genetic mouse models. For these studies, HDAC4/5 DKO [20] were used. These mice show several skeletal phenotypes due to abnormal osteocyte differentiation and function: high production of sclerostin, increased osteocyte density, and abnormally “woven” bone. The differences between osteocytes found in the calvaria of WT and HDAC4/5 DKO mice were studied with THG microscopy. THG optical sections consisting of 1 μm increments were obtained in order to visualize the osteocytes in 3D, and for analysis using Imaris (see Materials and methods).

**Fig 6:**
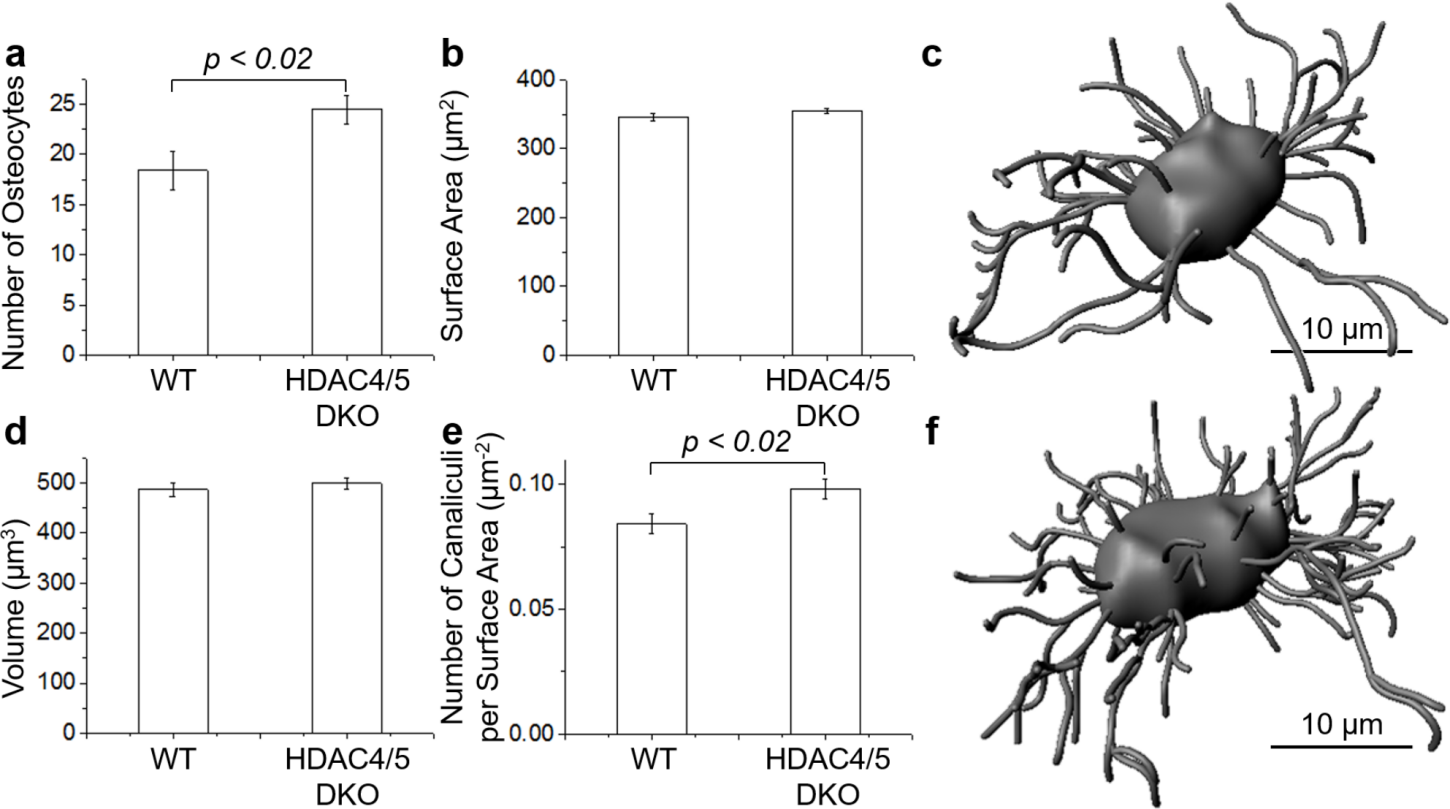
LCN parameters for WT and HDAC4/5 DKO mice. Measurement of the number of osteocytes in a volume consisting of 100 × 100 × 10 μm^3^ (a), lacunar surface area (b) and lacunar volume (d) per osteocyte as well as the number of canaliculi per lacunar surface area (e) in WT and HDAC4/5 DKO mice. Data are represented as the mean ± standard deviation (error bar). A statistically significant difference was not found in the lacunar surface area and volume measurements. The lacunar surface area, lacunar volume and number of canaliculi were determined from 3D renderings generated with Imaris software where an example osteocyte in WT (c) and HDAC4/5 DKO (f) mice is shown.

The number of osteocytes was counted in a volume consisting of 100 × 100 μm^2^ and within 10 μm of the surface of the bone. The investigation in 3 WT and 3 HDAC4/5 DKO mice revealed a statistically significant difference (*p* < 0.02, two-tailed T-test based on the number of mice studied) between the numbers of osteocytes in the two groups, as presented in Fig 6A. A mean of 18.4 ± 2.0 osteocytes and 24.5 ± 1.4 osteocytes was found in WT and HDAC4/5 DKO mice, respectively (where the error reported is the standard deviation). While the 3D data are in general agreement with previous results which showed HDAC4/5 DKO mice have significantly more osteocytes [20], the magnitudes of the variation is significantly different in THG versus histology. In a separate experiment, we compared the histology of 8 week old WT and HDAC4/5 DKO mice calvaria and confirmed the source of this discrepancy is not the bone type, where the calvaria of HDAC4/5 DKO mice had 2.8 ± 0.4 times more osteocytes than WT. More work is needed to understand the discrepancy in future studies to verify the THG microscopy results. One possible explanation is that we imaged significantly older mice (7 months old) than in reference [20], and the severity of the skeletal phenotype of these HDAC4/5 DKO animals could wane over time.

Surface area and volume measurements were made for a total of 60 osteocytes in 3 WT mice and 60 osteocytes in 3 HDAC4/5 DKO mice. Although the mean surface area and the mean volume of the lacunae of HDAC4/5 DKO mice is slightly larger than the mean surface area and the mean volume of the lacunae of WT mice (Fig 6B and 6D, respectively), a statistically significant difference between the two groups was not found.

The number of canaliculi per osteocyte in 3D were counted. A 3D reconstruction of the canaliculi was performed in Imaris, as a visual aid for counting (Fig 6C and 6F). Since the diameters of the canaliculi (95–550 nm [50]), are less than or equal to the resolution of the microscope (~0.5 μm lateral and ~3.0 axial), canaliculi size cannot be determined with the present microscope setup, and all the canaliculi are rendered as the same diameter. In order to normalize the data, the number of canaliculi per osteocyte was divided by its own surface area (Fig 6E). The result was 0.084 ± 0.004 μm^-2^ and 0.098 ± 0.004 μm^-2^ for WT and HDAC4/5 DKO mice, respectively (where the error reported is the standard deviation). A statistically significant difference (*p* < 0.02, two-tailed T-test based on the number of mice studied) between WT and HDAC4/5 DKO osteocytes was found, demonstrating there are more canaliculi per surface area present in the osteocytes of HDAC4/5 DKO mice versus WT mice. The number of canaliculi per surface area observed in this study is about a factor of 2 lower than what has been observed with confocal microscopy (~0.20 μm^-2^) using fixed and stained femur bone sections [50]. It is possible that osteocytes in different types of bones (calvarium versus femur) have different numbers of canaliculi, giving rise to the observed differences.

## Conclusions

THG microscopy with laser excitation wavelengths of 1550 nm to 1700 nm can be used for quantitative analysis of osteocytes in mouse calvarium *in vivo*. The THG signal originating from the bone-interstitial fluid boundary enables label-free imaging of the lacunae as well as the canaliculi. Both backward-directed and backscattered forward-THG contribute to the epi-collected THG image. Interestingly, the THG intensity from canaliculi that are tilted toward the optical axis have a higher intensity than canaliculi that are perpendicular to the optical axis. The intrinsic 3D imaging capability of nonlinear microscopy is used to investigate the number of osteocytes per volume, the lacunar volume and surface area as well as the number of canaliculi per lacunar surface area between WT and HDAC4/5 DKO mice. The study revealed statistically significant differences between the two mouse types in all the parameters investigated except the lacunar volume and surface area. Therefore, imaging osteocytes in the calvaria of mice with THG microscopy is a promising technique which can be used to effectively reveal differences in the structure of LCN in mice without the need for exogenous labels. Furthermore, THG microscopy allows the possibility of studying dynamical changes to the LCN during disease progression to better understand osteocytic regulation of bone.
